# Identify the Atrophy of Alzheimer’s Disease, Mild Cognitive Impairment and Normal Aging Using Morphometric MRI Analysis

**DOI:** 10.3389/fnagi.2016.00243

**Published:** 2016-10-18

**Authors:** Xiangyu Ma, Zhaoxia Li, Bin Jing, Han Liu, Dan Li, Haiyun Li, the Alzheimer’s Disease Neuroimaging Initiative

**Affiliations:** ^1^School of Biomedical Engineering, Capital Medical UniversityBeijing, China; ^2^School of Chinese Medicine, Capital Medical UniversityBeijing, China; ^3^College of Software Engineering, Beijing University of TechnologyBeijing, China

**Keywords:** MRI, Alzheimer’s disease, morphometric analysis, medial temporal lobe, gray matter volume, cortical thickness, atrophy indicator

## Abstract

Quantitatively assessing the medial temporal lobe (MTL) structures atrophy is vital for early diagnosis of Alzheimer’s disease (AD) and accurately tracking of the disease progression. Morphometry characteristics such as gray matter volume (GMV) and cortical thickness have been proved to be valuable measurements of brain atrophy. In this study, we proposed a morphometric MRI analysis based method to explore the cross-sectional differences and longitudinal changes of GMV and cortical thickness in patients with AD, MCI (mild cognitive impairment) and the normal elderly. High resolution 3D MRI data was obtained from ADNI database. SPM8 plus DARTEL was carried out for data preprocessing. Two kinds of *z*-score map were calculated to, respectively, reflect the GMV and cortical thickness decline compared with age-matched normal control database. A volume of interest (VOI) covering MTL structures was defined by group comparison. Within this VOI, GMV, and cortical thickness decline indicators were, respectively, defined as the mean of the negative *z*-scores and the sum of the normalized negative *z*-scores of the corresponding *z*-score map. Kruskal–Wallis test was applied to statistically identify group wise differences of the indicators. Support vector machines (SVM) based prediction was performed with a leave-one-out cross-validation design to evaluate the predictive accuracies of the indicators. Linear least squares estimation was utilized to assess the changing rate of the indicators for the three groups. Cross-sectional comparison of the baseline decline indicators revealed that the GMV and cortical thickness decline were more serious from NC, MCI to AD, with statistic significance. Using a multi-region based SVM model with the two indicators, the discrimination accuracy between AD and NC, MCI and NC, AD and MCI was 92.7, 91.7, and 78.4%, respectively. For three-way prediction, the accuracy was 74.6%. Furthermore, the proposed two indicators could also identify the atrophy rate differences among the three groups in longitudinal analysis. The proposed method could serve as an automatic and time-sparing approach for early diagnosis and tracking the progression of AD.

## Introduction

Alzheimer’s disease (AD) is an insidious onset neurodegenerative disease primarily characterized by progressive memory loss and accompanied by several kinds of cognitive and functional impairment (McKhann et al., 2011). Medial temporal lobe (MTL) structures such as hippocampus and entorhinal cortex are essential for declarative or long term memory, in which the AD core pathological changes and earliest atrophy takes place (Kimura et al., 2016). Mild cognitive impairment (MCI) can be regarded as a transitional period between normal aging and probable AD (Petersen et al., 2001). Several studies have proved that the atrophy of MTL structures are associated with the time to onset of MCI (Soldan et al., 2015) and the cognitively normal individuals with a greater rate of atrophy in MTL regions would potentially progress to MCI (Miller et al., 2013; Pacheco et al., 2015). Therefore, the atrophy in MTL structures may provide important information for MCI and early AD diagnosis and evaluating the risk of progression from normal to MCI and MCI to AD.

Numerous of studies have adopted morphometry information such as gray matter volume (GMV) and cortical thickness to detect the atrophy of MTL regions in AD and MCI. Reduction of hippocampal volume is a core biomarker for AD (Dubois et al., 2007, 2010; McKhann et al., 2011) and the severity of episodic memory deficits and cognitive disorders in MCI and AD are correlated with the hippocampal volume (Sarazin et al., 2010; McDonald et al., 2012). Cortical thinning was proved to be associated with MCI and poor episodic memory (Fujishima et al., 2014), and Pettigrew et al. (2016) found that normal cognitive individuals with low cortical thickness in AD vulnerable regions have a higher risk of progression to clinical symptom onset within 7 years of baseline. Moreover, the pattern of volume reduction of the hippocampal subfields combined with the cortical thinning of the adjacent extrahippocampal structures such as entorhinal and perirhinal cortex and parahippocampal cortex was found specific for AD compared with dementia with Lewy bodies (Delli Pizzi et al., 2016; Mak et al., 2016; Pettigrew et al., 2016). It suggests that the atrophy pattern characterized by the combination of GMV and cortical thickness of MTL structures may be useful to identify AD and MCI whose underlying pathophysiology is AD, and may overcome the specificity lacking for differentiating AD and MCI from other non-AD forms of dementia (Laakso et al., 1996; Chan et al., 2001; van de Pol et al., 2006; Bastos-Leite et al., 2007).

In order to acquire these atrophy morphometry informations from structural MRI brain images of patients, many morphometry methods were proposed. Manual hippocampus volumetry (Duara et al., 2008) is the gold standard to detect hippocampus atrophy but it is time consuming and costly (Pini et al., 2016). Alternatively, automatic methods such as surface based morphometry (SBM; Fischl, 2012) and voxel based morphometry (VBM; Ashburner and Friston, 2000) are preferred. Previously, surface based morphometric tools, for example, the Freesurfer software, is widely utilized in scientific studies, but need a long execution time. VBM has been widely used in the routine clinical due to time sparing as well as unbiased and comprehensive evaluation of structural differences throughout the brain (Matsuda, 2016). Recently, a VBM software named Morphometric Analysis Program (MAP) was developed and has been proved to be effective in detecting the focal cortical dysplasia (FCD) which is the possible lesion of epilepsy (Huppertz et al., 2005; Wagner et al., 2011). In the framework of this software, using SPM5 (Wellcome Department of Imaging Neuroscience, London, UK) for data preprocessing, feature maps called ‘extension image’ and ‘thickness image’ were calculated via voxel wise *z*-score analysis to highlight the subtle abnormality of gray matter density (GMD) and cortical thickness of the epilepsy patients compared with a normal controls database (NCDB).

High registration quality is very important for voxel-by-voxel *z*-score analysis to obtain reliable *z*-score maps. However, the quality of registration only using old versions of SPM might be insufficient to buttress a convincing *z*-score analysis to reveal the subtle changes between the cognition impaired patients and the normal healthy or tracking the gradual process of brain atrophy. Recent years, an algorithm called diffeomorphic anatomical registration using exponentiated Lie algebra (DARTEL) (Ashburner, 2007) has been available in new versions of SPM (SPM8 and SPM12). DARTEL has stronger ability to deal with the local anatomical differences among individuals, thus, to achieve higher registration accuracy. In a previous VBM study (Matsuda et al., 2012) using SPM8 plus DARTEL, *z*-score analysis was carried out to estimate the GMV decline, and several indicators were proposed base on the *z*-score map to discriminate very mild AD patients from normal controls and reached considerable accuracy.

In this study, based on the MAP framework, we proposed a modified morphometric MRI analysis method to quantitatively assessed the GMV and cortical thickness decline in MTL structures of AD, MCI, and normal control (NC), and to validate the hypothesis that the degrees and rates of atrophy in MTL of AD, MCI and normal aging are different, from severe to slight. Both cross-sectional and longitudinal atrophy characteristics are taken into consideration.

## Materials and Methods

### Data Acquisition

The subject data used in this study was downloaded from the public Alzheimer’s Disease Neuroimaging Initiative (ADNI) database^1^. The ADNI was launched in 2003 as a public-private partnership, led by Principal Investigator Michael W. Weiner, MD. ADNI aims to seek sensitive and accurate methods and biomarkers to serve MCI and AD early diagnostic, as well as to track the progress of the disease and evaluate the effects of treatment or potential interventions. The ADNI database provide abundant and quality assured structural MRI data of people without memory problems, patients with MCI and patients who had been diagnosed AD. The inclusion/exclusion criteria are as follows (for up-to-date information^2^):

(1) Normal subjects: MMSE scores between 24 and 30 (inclusive), a CDR of 0, non-depressed, non-MCI, and non-demented. The age range of normal subjects will be roughly matched to that of MCI and AD subjects.
(2) MCI subjects: MMSE scores between 24 and 30 (inclusive), a memory complaint, have objective memory loss measured by education adjusted scores on Wechsler Memory Scale Logical Memory II, a CDR of 0.5, absence of significant levels of impairment in other cognitive domains, essentially preserved activities of daily living, and an absence of dementia.
(3) AD subjects: MMSE scores less than 24 (inclusive), a CDR greater than 0.5 (inclusive), and meets NINCDS/ADRDA criteria for probable AD (this criteria is different from the one in ADNI site for this study).
(4) At least 1 year and three visits structural MRI data is available.

A total of 88 subjects (15 AD, 23 MCI, and 51 NC) with 1–4 years follow-up (3–7 visits) MRI data were finally included in this study. According to the MAP framework, we randomly selected 25 NC from the 51 NC to be the NCDB to establish a normal distribution for *z*-score analysis. Atrophy characteristics computation was performed among the 15 AD, 23 MCI, and the remaining 26 NC. The baseline information of the subjects is shown in **Table 1**.

**Table 1:** Descriptive baseline statistical information for the subjects.

Group	AD	MCI	NC	NCDB
Sample size	15	23	26	25
Age^a^	74.5 ± 8.5	73.4 ± 9.0	76.1 ± 8.1	73.8 ± 6.6
Female percentage^b^	53%	39%	54%	52%
MMSE^c^	21.5 ± 3.4	25.4 ± 3.3	28.8 ± 1.2	28.9 ± 1.2

*MMSE, mini-mental state examination.*

^a^*One-way ANOVA among groups: *F* = 0.308, *p* = 0.819.*

^b^*χ^*2*^ test for gender composition among groups: χ^*2*^= 1.336, *p* = 0.721.*

^c^*One-way ANOVA among groups: *F* = 50.14, *p* < 0.001; Turkey’s pairwise comparison: AD vs. MCI, AD vs. NC, AD vs. NCDB, MCI vs. NC, MCI vs. NCDB, *p* < 0.001; NC vs. NCDB, *p* = 0.98*.

All the subjects were scanned by Trio-Tim 3 Tesla MRI System (Siemens, Erlangen, Germany). High resolution T1-weighted magnetization prepared rapid gradient echo (MPRAGE) images (voxel size: 1 mm × 1 mm × 1.2 mm, image size: 240 × 256 × 176 voxel) were obtained with the following parameters: TE = 2.95 ms, TR = 2300 ms, TI = 900 ms, flip angle = 9°.

### Data Preprocessing

All the 3D T1-weighted images were intensity corrected and unified segmented by SPM8, and rigidly aligned tissue classes images (gray matter images and whit matter images, voxel size: 1 mm × 1 mm × 1 mm) were obtained. DARTEL was adopted to generate a series of increasingly crisp customized templates using the gray matter and white matter images of NCDB.

The gray matter and white matter images of all the AD and MCI patients together with the remaining 26 normal controls were non-linearly and iteratively registered to the increasingly crisp customized templates by DARTEL. Then the aligned gray matter images were normalized to the MNI space followed by modulation to preserve the amount of gray matter. After that, global GMV normalization was done to correct the inter-subject variance of the total brain volume.

### GMV *z*-Score Map Calculation

The modulated and normalized gray matter images (including the images of NCDB) were smoothed by a 6 mm full width at half maximum (FWHM) Gaussian kernel. Then, a mean image and a standard deviation image were generated by computing the mean and standard deviation of the corresponding voxel values in the smoothed gray matter images of NCDB. Voxel-by-voxel *z*-score was calculated for the smoothed gray matter images of the three groups so that we obtained GMV *z*-score map for each individual. Here, the voxel wise *z*-score was defined as:

$$Z=\left(X-{\bar{X}}_{N}\right)/\sigma_{N}\,\;\;\;\;\;\;\;\;\;\;\;\;\;\;\;\;\;\;\;\;\;\;\;\;\;\;\;\;\;\;\;\left(1\right)$$

where *X* was the voxel value of an individual, ${\bar{X}}_{N}$ and σ_N_ was the corresponding voxel value of the mean and standard deviation image of NCDB, respectively.

### Cortical Thickness *z*-Score Map Calculation

The modulated and normalized gray matter images (including the images of NCDB, not smoothed here) were converted to binary images with a threshold of 0.4. Run-length vectors were computed for each voxel in the gray matter (the voxels with value 1 in the binary image). These vectors were determined along 26 spatial directions from the starting voxel to the tissue boundary. The Euclidean lengths of each pair of opposing vectors were summed, and the minimum of the 13 values was adopted to be the run-length of the starting voxel. The run-lengths of the voxels not in the gray matter were set to 0. Then the standard deviation run-length image of the NCDB was smoothed by a 3 mm FWHM Gaussian kernel to avoid very large or even infinite negative *z*-score. After that, a similar *z*-score comparison analysis was carried out to acquire cortical thickness *z*-score map for each individual. A difference from GMV *z*-score map calculation was that the cortical thickness *z*-score was only calculated for the voxels in the gray matter of each individual.

### VOI Definition and Atrophy Indicators Calculation

Following the method proposed by Matsuda et al. (2012), we defined a VOI via group comparison between the GMV images of the AD patients and the normal controls. The resulting region contained bilateral MTL structures such as hippocampus, amygdala, and entorhinal cortex, which was consistent with the region reported in Ref. 12. GMV decline indicator was defined as the mean of the negative *z*-scores in the VOI. We used the same VOI for cortical thickness decline indicator calculation, because abnormal GMV decrease may accompanied by abnormal cortical thinning. The cortical thickness decline indicator was defined as the sum of the normalized negative *z*-scores in the VOI. Here, the normalization means that the original *z*-score of each voxel was divided by the corresponding run-length of the voxel itself.

### Whole VOI Based Prediction with Support Vector Machines (SVM)

To evaluate the predictive performance atrophy indicators, i.e., the ability of them to identify the label of unknown subjects, we implemented a support vector machines (SVM) based prediction with a leave-one-out cross-validation design using the baseline atrophy indicators. This procedure was carried out by LibSVM (Chang and Lin, 2011)^3^. Both two-way and three-way predictions were taken into considered.

The atrophy indicators of all the subjects were normalized by z-transformation and the resulting atrophy features were inputted into a SVM with RBF kernel. For each of the two indicators and the combination of them, we separately applied a leave-one-out cross-validation (LOOCV) and grid search based procedure to find the optimal regularization factor and RBF kernel parameter for the SVM model. The predictive accuracy was defined as the percentage of the correctly predicted cases in LOOCV with the optimal SVM model.

### Multi-Region Based Prediction with SVM

With the similar feature calculation method for the whole VOI, we calculated the atrophy features in six sub-regions of the VOI, i.e., VOI-covered bilateral hippocampus, amygdala, and parahippocampal gyrus. These sub-regions were obtained by comparing the VOI with Automated Anatomical Labeling – 90 (AAL-90) (Tzourio-Mazoyer et al., 2002) template. Then, we applied a similar SVM based prediction procedure to evaluate the predictive performance of the indicators.

### Estimation of the Indicators’ Changing Rates

Using the presented method, we calculated GMV and cortical thickness decline indictors for all the follow-up data of the subjects. Then we adopted linear least squares estimation (LLSE) to assess the changing rates of the indicators as estimation of atrophy rate.

### Evaluation of the Correlation between the Atrophy Indicators

The correlation between the GMV and cortical thickness indictors was estimated by Spearman rank correlation coefficients due to the non-normality of the data.

### Statistical Method

Kruskal–Wallis *H* tests were applied to examine the differences of the atrophy indicators among the three groups, and if significant difference was found by *H* test, Mann–Whitney *U* tests would be applied to make pairwise comparisons and Holm–Bonferroni correction was used to determine if the *post hoc* tests are significant.

## Results

### Baseline GMV and Cortical Thickness *z*-Score Maps and Atrophy Indicators

**FIGURE 1:**
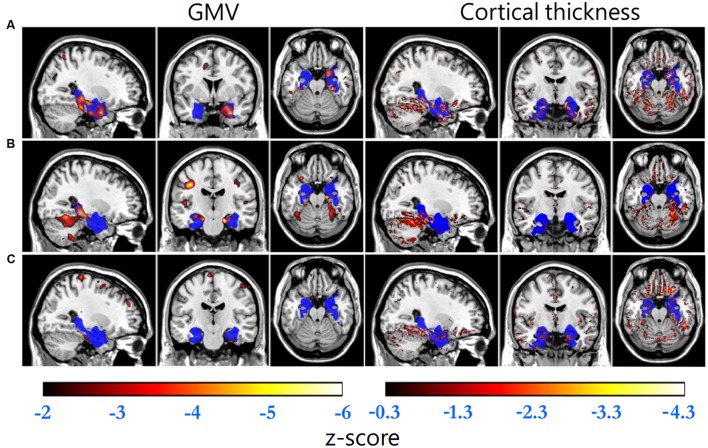
**Standard brain template overlaid by the VOI (blue) and the regions with GMV *z*-scores less than -2 (hot, left column) and cortical thickness *z*-scores less than -0.3 (hot, right column) of **(A)** an AD patient (GMV decline indicator: -1.699, cortical thickness decline indicator: -0.079), **(B)** a MCI patient (-1.122, -0.011) and **(C)** a normal control (-0.502, -0.013), respectively**.

Gray matter volume and cortical thickness *z*-score maps of an AD, a MCI and a NC subject were shown in **Figure 1**. We could tell significant differences of atrophy degree and atrophy regions among the AD, MCI, and NC subjects according to the *z*-score maps. The AD patient showed obvious GMV loss and cortical thinning in right hippocampus and entorhinal cortex. The MCI patient also showed GMV decline in bilateral hippocampus and right entorhinal cortex, however, with a much smaller GMV decline area and slight cortical thinning in VOI. For the normal control, slight cortical thinning and almost no abnormal decrease of GMV were found with the *z*-score maps.

**FIGURE 2:**
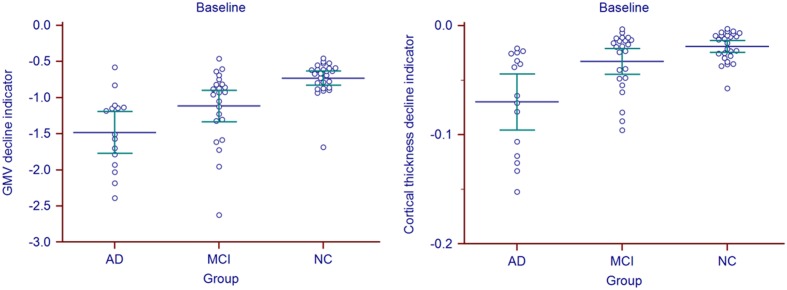
**Baseline atrophy indicators for all the individuals in AD, MCI, and NC group**.

Statistic results of the baseline atrophy indicators were listed in **Table 2** (left) and the atrophy indicators value of all the individuals were shown in **Figure 2**. AD group had significant smaller GMV decline indicator (*p* = 0.0282, corrected) and cortical thickness decline indicator (*p* = 0.0138, corrected) than MCI group. MCI group had significant smaller GMV decline indicator (*p* = 0.0009, corrected) and cortical thickness decline indicator (*p* = 0.0473, corrected) than NC group. Also, these two indicators of AD group were both significantly smaller than NC group (*p* < 0.0001 for GMV and *p* = 0.0002 for cortical thickness, corrected).

**Table 2:** Statistic results of the baseline atrophy indicators and their longitudinal changing rate for AD, MCI, and NC group.

	Group	Atrophy indicators at baseline		Atrophy indicators’ changing rate (per year)	
		GMV	Cortical thickness	GMV	Cortical thickness
Value (mean ±*SD*)	AD	-1.487 ± 0.520	-0.074 ± 0.053	-0.092 ± 0.090	-0.012 ± 0.007
	MCI	-1.121 ± 0.505	-0.032 ± 0.024	-0.056 ± 0.053	-0.005 ± 0.003
	NC	-0.733 ± 0.240	-0.019 ± 0.012	-0.030 ± 0.044	-0.003 ± 0.003
Kruskal–Wallis test^a^		H = 24.33, ***p* < 0.0001**	H = 18.34, ***p* = 0.0002**	H = 8.25, ***p* = 0.0160**	H = 19.43, ***p* < 0.0001**
Pairwise *post hoc* test^a,b^	AD vs. MCI	***p* = 0.0282**	***p* = 0.0138**	*p* = 0.1618	***p* = 0.0006**
	AD vs. NC	***p* < 0.0001**	***p* = 0.0002**	***p* = 0.0178**	***p* < 0.0001**
	MCI vs. NC	***p* = 0.0009**	***p* = 0.0473**	*p* = 0.1618	***p* = 0.0045**

*AD, Alzheimer’s disease; MCI, mild cognitive impairment; NC, normal control; GMV, gray matter volume; SD, standard deviation.*

^a^*The *p*-values were adjusted by Holm–Bonferroni method. Null hypotheses (no significant difference) were rejected at level α = 0.05.*

^b^*Mann–Whitney *U* test for pairwise comparison. Boldfaced characters represent the statistically significant results*.

### Predictive Accuracy of the Indicators

The predictive accuracies of the VOI based and multi-region based SVM models with one kind (only GMV or only cortical thickness) and two kinds of (GMV + cortical thickness) decline indicator were listed in **Table 3**.

**Table 3:** Predictive accuracies of the atrophy indicators.

	Whole VOI based			Multi-region based		
Decline indicator	GMV	Cortical thickness	GMV+ Cortical thickness	GMV	Cortical thickness	GMV+ Cortical thickness
AD vs. NC	92.7%	80.5%	92.7%	92.7%	90.2%	92.7%
MCI vs. NC	87.5%	75.0%	87.5%	87.5%	77.1%	91.7%
AD vs. MCI	67.6%	78.4%	78.4%	70.3%	78.4%	78.4%
Three-way^∗^	69.8%	65.1%	71.4%	74.6%	65.1%	74.6%

*^∗^Three-way, Three-way prediction*.

### Longitudinal Changing Rate of the Atrophy Indicators

**FIGURE 3:**
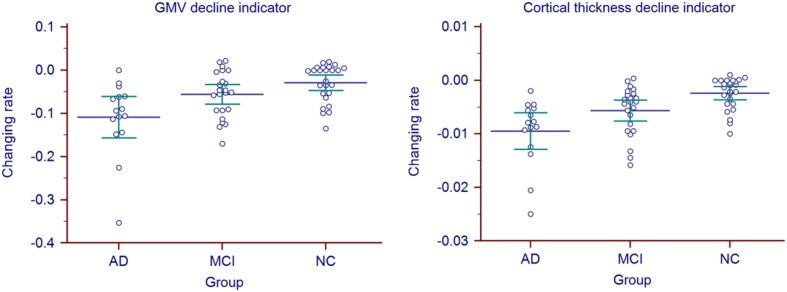
**Atrophy indicators’ longitudinal changing rate of all the individuals in AD, MCI, and NC group**.

Longitudinal changing rate of the atrophy indicators (**Table 2**, right) were significantly different between the AD and NC group (*p* = 0.0178 for GMV, *p* < 0.0001 for cortical thickness, corrected). With cortical thickness decline indicator changing rate, we could also significantly differentiate MCI group from the AD (*p* = 0.0006, corrected) and NC (*p* = 0.0045, corrected). However, with the GMV decline indicator changing rate, no statistic significant difference was found between MCI group and the other two groups (both with *p* = 0.1618, corrected). The longitudinal changing rate of the atrophy indicators of all the individuals were shown in **Figure 3**.

### Correlation between the Atrophy Indicators

Significant correlation was only found between the changing rates (*r*_S_ = 0.726, *p* = 0.0006, corrected) of the two atrophy indicators in MCI group.

## Discussion

In this study, we established an improved morphometric MRI analysis method based on MAP framework to quantitatively evaluate brain atrophy. This method uses SPM8 and DARTEL for data preprocessing. Significant atrophy degree and rate differences among the three groups (follow the order of AD > MCI > NC) could be identified by the proposed GMV and cortical thickness decline indicators. Moreover, the predictive performance of the proposed indicators was promising.

The proposed method was implemented using MATLAB R2012a (The Mathworks, Inc., Natick, MA, USA) under Microsoft Windows 10 64-bit operating system. The average execution time was measured on an Intel 2.5 GHz machine with 8 GB RAM. It takes about 15 min to process the MRI data of an individual from data preprocessing to obtaining the GMV and cortical thickness decline indicators, and the whole procedure was carried out automatically with MATLAB scripts. As the processing time is much shorter than that of the Freesurfer pipelines which are widely used in scientific researches, this method has the potential to be adopted in routine clinic.

The main difference between MAP and the presented method is that the region of interest of MAP was highlighted by large positive *z*-scores, which indicates the gray matter extends abnormally into the white matter and the abnormal thick cortex. However, this study was interested in the regions with negative *z*-scores which indicate the gray matter loss and abnormal thin cortex. In addition, MAP used unmodulated gray matter images, i.e., gray matter density (GMD) images to calculate the *z*-score map, while we used modulated, i.e., GMV images followed by global GMV normalization for we wanted to compare the absolute amount of gray matter to quantitatively assess the gray matter loss.

For cortical thickness *z*-score map of each individual, in order to avoid negative *z*-scores at the voxels out of the gray matter which not directly reflect the abnormality between the individual’s real cortical thickness and the normal thickness and might be confounded by a inter-individual tissue boundary variance which is not related to cortical thinning, we only calculated *z*-scores for the voxels in the gray matter, i.e., voxels with non-zero value in the binary image. In this condition, we cannot simply take the mean of the negative *z*-scores in the VOI as the cortical thickness decline indicator, because when the mild atrophy regions containing small *z*-scores and the server atrophy regions containing large both exist in the VOI, the average *z*-values will underestimate the actual degree of atrophy of the VOI. Hence, we proposed to use normalized *z*-scores to replace the original *z*-scores, that is, the *z*-score of each voxel was divided by the corresponding run-length at the voxel itself. Thus, the contribution of serious cortical thinning to the cortical thickness decline indicator could be enhanced in contrast that the contribution of slight thinning could be weakened.

In MAP, the standard deviation image of the NCDB used for *z*-score analysis was smoothed by a 6 mm FWHM Gaussian kernel to avoid outliers at the positions where the standard values are too small or zero. This is because few or no normal controls contribute to the NCDB mean and standard deviation at these positions (i.e., most or all of the normal controls in NCDB have zero value here). In this study, to acquire more precise assessment of atrophy, we didn’t smooth the standard deviation image of the NCDB for the *z*-score analysis for GMV because the regions described above can only exist at the border of the standard brain, which is far from the VOI at the MTL. Meanwhile, we used a Gaussian kernel with smaller FWHM (3 mm, compared with the 6 mm FWHM kernel used in MAP) to smooth standard deviation image of NCDB for cortical thickness, as there might be several voxels in the standard deviation image having zero values (i.e., all the subjects of NCDB have the same run-length there), which may cause infinite *z*-scores thus necessitate the smoothing. The use of a smaller FWHM kernel is important for correctly assessing the cortical thickness change in spindly or small structures such as hippocampus, entorhinal cortex, and amygdala, and for enhancing the difference of cortical thinning between patients and normal controls.

In this paper, the VOI determined by comparison between AD and NC group widely covers hippocampal and adjacent extrahippocampal regions in MTL, which are closely related to AD pathology. Among these regions, hippocampus atrophy is the best established and validated one for staging the progression AD pathology (Jack et al., 2011) and the atrophy process correlated with clinical decline (Fox et al., 1996). The reduction of hippocampal volume of clinical AD patients range from 15 to 40% compared with NC (Bosscher and Scheltens, 2002) while only by 10–15% for MCI (Shi et al., 2009). Entorhinal cortex is the connection point of the hippocampus and neocortex, in which early neurofibrillary tangles and tau deposits arise (Braak and Braak, 1995; Braak and Del Tredici, 2015). Many studies have demonstrated a more serious entorhinal cortex volume and thickness reduction in AD than NC (Pennanen et al., 2004; Teipel et al., 2006; Velayudhan et al., 2013), and the degree of EC volume reduction in MCI was proved to be between that in NC and AD (Stoub et al., 2006; Devanand et al., 2012; Velayudhan et al., 2013). Besides, amygdala has abundant neural connections with the hippocampus, and it is another region that early affected by neurofibrillary tangles formation in AD. The reduction of amygdala volume may range from 15–20% to 33–37% with AD progression (Pini et al., 2016). In this study, atrophy indicators calculated in VOI can reflect the overall atrophy situation in these regions. As expected, the mean values of the GMV and cortical thickness decline indicators of MCI group both fall in between the AD and NC group. This demonstrates that MCI is a transitional period between normal aging and AD. In addition, AD group showed large intra-group atrophy variance, which may due to the different atrophy severities of patients in different AD stages.

Gray matter volume reduction in cortices causes either cortical area loss or cortical thinning. The correlation between GMV and cortical thickness decline indicators may reflect the proportion of cortical thinning accompanied GMV reduction. In other words, strong correlation may represent that GMV reduction mainly causes cortical thinning rather than cortical area loss. According to the results in **Table 4**, we can find that: (1) The correlation between the two atrophy indicators at the baseline and between their changing rates are both non-significant in NC group. (2) The correlation between the changing rates of the two indicators in MCI group is the strongest. (3) At the baseline, although with no statistical significance, the correlation of the two indicators in AD group tends to be the strongest. Reasonable explanations are the follows: For normal aging, the cortical area loss and cortical thinning may both exist in MTL and the extent and rate of these two processes are both small thus poor correlation could be observed between the GMV and cortical thickness decline [matching finding (1)]. In MCI period, gray matter loss may mainly causes cortical thinning while the cortical area loss may be relatively inconspicuous [matching finding (2)]. With the accumulation of the cortical thinning accompanied gray matter loss, the correlation between cortical thickness and GMV decline become more prominent [matching finding (3)], and this process may corresponding to the progression from MCI to AD. In diagnosed AD stage, the atrophy can be very severe and both cortical area loss and cortical thinning are conspicuous, so the correlation between the changing rates of the two indicators is weaken [matching finding (2)]. The reason why statistical significance was only found between the changing rates of the two atrophy indicators in MCI group might be the small sample size (especially for AD group). The correlation between the two indicators and between their changing rates remains to be investigated in future study with larger sample size.

**Table 4:** Correlation between the baseline GMV and cortical thickness decline indicators (left column), and their longitudinal changing rate (right column) for AD, MCI, and NC group.

	Spearman rank correlation coefficients^∗^	
	Baseline	Longitudinal changing rate
AD	0.569 (*p* = 0.1134)	0.593 (*p* = 0.1188)
MCI	0.407 (*p* = 0.3234)	**0.726** **(*p* = 0.0006)**
NC	0.197 (*p* = 1.0000)	0.295 (*p* = 0.9156)

*^∗^*P*-values of the correlation coefficients were corrected by Bonferroni method. Boldfaced characters represent the statistically significant results*.

The GMV decline indicator investigated in this study is similar to an indicator proposed by Matsuda et al. (2012) which was integrated in a voxel-based specific regional analysis system for AD (VSRAD). In their study, through a ROC analysis with the ‘atrophy severity in VOI’ indicator, the discriminative accuracy of very mild AD from NC was up to 91.6%. In our study, the predictive accuracies obtained from LOOCV for AD and NC discrimination and MCI and NC discrimination were 92.7 and 87.5%, which demonstrated the good discrimination performance of the GMV as a biomarker not only for differentiating AD from NC, but also for differentiating MCI from NC. In VSRAD, another indicator called ‘extent in target VOI’ was defined as the percentage of voxels with *z*-score less than -2 in the VOI. We calculated this indicator based on the GMV *z*-score maps obtained in this study and found this indicator was highly correlated (*r* = 0.98, *p* < 0.0001) to the GMV decline indicator. This high correlation may due to the smoothing process in GMV *z*-score map calculation.

The cortical thickness decline indicator newly proposed in this study gave a relatively low predictive accuracy (80.5, 75.0%) than GMV (92.7, 87.5%) in differentiating AD from NC and MCI from NC. This may arise from that some cognitive normal individuals also have low cortical thickness in MTL (Pettigrew et al., 2016). On the other hand, the cortical thickness decline indicator showed higher accuracy (78.4%) than GMV (67.6%) in differentiating AD from MCI. This suggest that cortical thickness is a more sensitive marker for identify the atrophy difference between AD and MCI. As the two indicators both have their own advantages, alternative to the single indicator prediction, we used the combination of these two indicators to make predictions and achieved generally higher accuracies (The forth column in **Table 3**).

The use of multi-region based prediction model was more accurate than the whole VOI based model and we acquired the highest general predictive accuracy (92.7% for AD vs. NC, 91,7% for MCI vs. NC, 78.4% for AD vs. MCI and 74.6% for three-way prediction) by applying multi-region based prediction model with the combination of the two atrophy indicators. This is because the spatial distribution of the atrophy is also important for identifying the atrophy patterns. Hippocampus and adjacent extrahippocampal structures are consisted of several subfields, but accurately segmentation of these subfields remains a challenge. Hence, the brain regions (hippocampus, parahippocampal gyrus, and amygdala) selection in this study is based on AAL-90 atlas, which is a widely used whole brain atlas that doesn’t label these subfields. Recent MRI studies (Mueller and Weiner, 2009; Tang et al., 2015; Delli Pizzi et al., 2016; Mak et al., 2016) revealed that AD pathology would differently affects the hippocampal subfields, i.e., subiculum, cornu ammonis sectors (CA) 1–3 and dentate gyrus (Duvernoy, 2013) and increasing evidence suggested that the prediction sensitivity and accuracy of pathological alterations the hippocampal subfields than the whole hippocampus (Apostolova et al., 2010; Maruszak and Thuret, 2014). Besides, impairment in adjacent extrahippocampal subfield such as entorhinal, perirhinal, and parahippocampal cortices also contributes to typical deficit in episodic memory, which is the earliest characteristic of AD (Nadel and Hardt, 2011). In future study, the proposed method is expected to reveal pathology specific atrophy patterns of these subfields using more sophisticated atlas. Furthermore, since our method can give unbiased evaluation of abnormal changes throughout the whole brain, alternations in neocortical cortex such as parietal and frontal lobe cortex and subcortical structures such as amygdala can also be utilized to track and predict the progression of the disease.

The MRI data used in this study was acquired by the same kind of scanner (Siemens TrioTim 3T) and with the same scanning parameters. However, MRI scanners of different manufacturers may have different inner image modifying techniques, thus may cause bias when we use *z*-score analysis to compare new patients’ MRI data with a previously established NCDB based on a different kind of scanner or parameters. Therefore, more validation study for the reproducibility of this method for multiple center data and the statistics study for correcting the indicator variance between different kinds of scanner and parameters are desired. Before a systematic study to prove the reproducibility of this method among different kinds of scanner and scanning parameters, a good solution is that AD centers can establish several NCDBs for different MRI scanners they own, and analysis new patients’ data based on the corresponding NCDB.

## Author Contributions

XM and ZL made substantial contributions to the conception, design, analysis and interpretation of data, and has drafted the manuscript. BJ and HLiu contributions to the design of the work and revision of the manuscript. DL made contributions to collection of data. HLi, the corresponding author, made contributions to conception and interpretation of data, have determined the final version to be submitted for publishing. All authors read and approved the final manuscript.

## Conflict of Interest Statement

The authors declare that the research was conducted in the absence of any commercial or financial relationships that could be construed as a potential conflict of interest.

## Footnotes

1 http://adni.loni.usc.edu

2 www.adni-info.org

3 http://www.csie.ntu.edu.tw/~cjlin/libsvm
